# Menstrual health and Attention-Deficit/Hyperactivity Disorder (ADHD) symptoms: A scoping review

**DOI:** 10.1177/17455057261460285

**Published:** 2026-06-11

**Authors:** Gabriela Kennedy, Megan Baran-Goldwax, Sarah Lippé

**Affiliations:** 1 Department of Psychology, 192306 University of Montreal, Montreal, QC, Canada; 2 CHU Sainte-Justine Research Centre, 70443 University of Montreal, Montreal, QC, Canada

**Keywords:** ADHD, attention-deficit/hyperactivity disorder, menstruation, menstrual health, dysmenorrhea, neurodevelopment

## Abstract

**Background:**

Little is known about how menstrual health characteristics may influence Attention-Deficit/Hyperactivity Disorder (ADHD) symptoms, despite evidence that hormonal fluctuations can affect attention, mood, and behaviour.

**Objective:**

This scoping review examined the extent, nature, and quality of the evidence exploring the relationship between menstrual health features and ADHD symptom presentation.

**Eligibility Criteria:**

All primary study designs were considered. Studies were included if they investigated ADHD diagnosis or symptoms in relation to menstrual health factors. Reviews, commentaries, theses, and conference abstracts were excluded.

**Sources of Evidence:**

A structured, keyword-based search was conducted on MEDLINE (Ovid), PsycINFO (Ovid), and Web of Science on July 18th, 2025. Titles, abstracts, and full texts were screened in Covidence.

**Charting Methods:**

Data were extracted using a standardized form and synthesized narratively according to themes identified during data extraction. A critical appraisal of included studies was conducted using an adapted risk of bias tool.

**Results:**

The search yielded 691 records. 20 studies met inclusion criteria. Most were published after 2020 and employed non-randomized experimental, cross-sectional, qualitative, or case study designs. Sample sizes ranged from 1-405, with participants aged 13-49. Six themes emerged: (1) comorbid ADHD symptoms in individuals with menstruation-related disorders; (2) menstrual difficulties and ADHD symptoms; (3) menstrual phase-related worsening of ADHD symptoms; (4) menstrual side-effects of stimulant medication; (5) menstrual pain-related attentional interference; and (6) menstruation-tailored interventions. Quality appraisal indicated that half of the studies were rated Very Good. Gaps in reporting sample size justification and measures of gender identity were common.

**Conclusions:**

Emerging evidence links menstrual health and ADHD symptoms. Despite growing interest, the field is marked by small samples, heterogeneous methods, and limited attention to gender identity. Future research should justify sample sizes, integrate gender-sensitive measures, and further explore interventions tailored to menstruation-related ADHD symptom fluctuations.

## Plain Language Summary

Hormonal changes throughout the menstrual cycle are known to affect the brain, but little is understood about how they influence Attention-Deficit/Hyperactivity Disorder (ADHD) symptoms. To learn more, we reviewed all published research on the connection between menstrual health and ADHD. We searched three major scientific databases in July 2025 and found 691 studies. After screening, 20 studies were included. These studies involved people aged 13-49 and used multiple types of research methods, such as experiments, surveys, interviews, and case reports. From these studies, six main patterns emerged: (1) ADHD symptoms in people with other menstrual health conditions; (2) Links between painful, heavy, and irregular periods and ADHD symptoms; (3) Worsening of ADHD symptoms at different phases of the menstrual cycle; (4) Menstrual side effects of certain ADHD medications (e.g., Concerta); (5) Attention problems linked to menstrual pain; (6) Early trials of ADHD interventions tailored to menstrual cycles. Half of the studies were rated as very good quality. Many had small sample sizes and did not report participants’ gender identity or explain how they decided on the number of people to include. Overall, this review shows growing evidence that menstrual health and ADHD symptoms may be connected. More rigorous and inclusive research is needed to better understand these links and to design interventions that help people manage ADHD symptoms that change across the menstrual cycle.

## Background

### Attention-deficit/hyperactivity disorder

Attention-Deficit/Hyperactivity Disorder (ADHD) is a neurodevelopmental condition characterized by developmental differences in three core symptom domains: inattention, hyperactivity, and impulsivity.^1,2^ ADHD is categorized into three subtypes, Inattentive, Hyperactive/Impulsive, and Combined, which are defined by differing patterns of these three symptoms.^3^ The Inattentive subtype is characterized primarily by symptoms of inattention, including difficulty sustaining attention, disorganization, and distractibility.^3^ The Hyperactive/Impulsive subtype is characterized by hyperactivity, such as fidgeting, excessive talking, and difficulty remaining still, as well as impulsivity, including difficulty waiting one’s turn and interrupting or intruding on others.^3^ The Combined subtype refers to symptom presentations that incorporate both inattentive and hyperactive-impulsive symptoms.^3^

Symptoms associated with ADHD are common in the general population, with only a small proportion of individuals meeting diagnostic criteria for the disorder. ADHD is the most prevalent psychiatric disorder in children,^4^ affecting 8% of youth worldwide.^5^ ADHD symptoms often persist into adulthood,^2^ with 6.76% of adults remaining symptomatic.^2^ Beyond its impact on children’s everyday lives and developmental trajectories,^2^ emerging work has found that ADHD can be associated with significant familial and societal economic burden.^5,6^ Early and accurate identification across diverse populations and symptom presentations is essential to improve developmental outcomes^7^ and to alleviate the wider familial and societal impacts that arise from underdiagnosis and inadequate support.^6^

### Menstrual health and ADHD symptoms

There is substantial evidence that biological sex and gender identity may shape the presentation and experience of ADHD symptoms.^8,9^ While the existing literature on ADHD symptoms in individuals assigned female at birth (AFAB) has largely focused on developmental trajectories over time,^10–12^ there may be sex-specific hormonal changes that contribute to variations in symptoms on a shorter timescale. That is, the development of the menstrual cycle may influence disorder symptomatology on a monthly, recurring basis. The menstrual cycle refers to the female reproductive system’s cyclical preparation for ovulation and potential pregnancy, typically lasting 24 to 38 days, and consisting of four phases: the menstrual phase, the follicular phase, the ovulatory phase, and the luteal phase.^13,14^ These four phases are often collapsed into two broader categories: follicular (encompassing menstrual and ovulatory phases) and luteal.^15^ For the sake of precision, however, the present review will refer to the menstrual cycle in four distinct phases.

**Figure 1.**
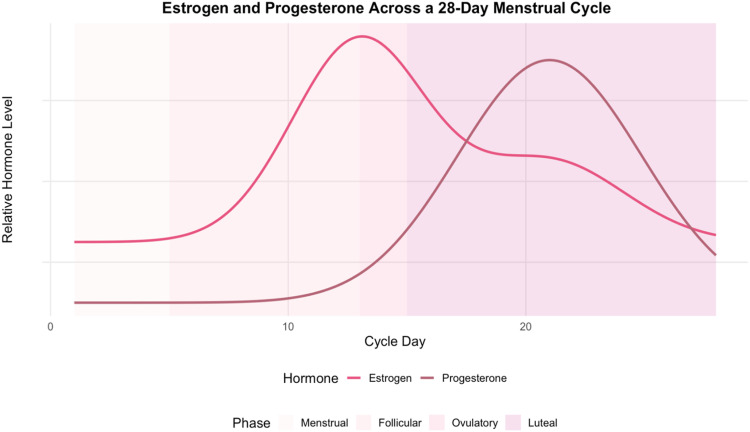
Estrogen and progesterone across a 28-day menstrual cycle. *Note.* This is a conceptual representation. Values are intended for illustration and do not reflect exact measurements.

It is important to note that Figure 1 and the following description are a “typical/regular” account of the 28-day menstrual cycle and do not capture individual variability in cycle length due to factors like stress,^16^ body mass index (BMI),^17^ age at menarche,^18^ and other factors that may influence menstrual cycle length. The beginning of the menstrual cycle is marked by the shedding of the uterine lining (i.e. menstruation), which typically lasts 3 to 7 days.^14,15^ During the menstrual phase, estrogen and progesterone levels are at their lowest.^14^ Following menstruation, the follicular phase begins, with estrogen gradually rising over approximately 7 to 15 days and culminating in a peak that triggers ovulation (at around day 14-19 in a 28-day cycle).^19^ After ovulation, the luteal phase, which lasts 14 days during a 28-day cycle^15^ is marked by a decline in estrogen, as well as a rise and subsequent decline in progesterone levels before menstruation.^14,15^ This phase signals the end of the menstrual cycle, with declining estrogen and progesterone eventually initiating the next menstruation.^14^

Many individuals also experience menstrual differences that may alter their experience of menstruation. These differences can be observed in cycle length, bleeding severity, and menstrual pain, among other features. For example, some individuals may experience severe menstrual pain (dysmenorrhea), heavy menstrual bleeding (menorrhagia), prolonged menstrual bleeding (hypermenorrhea), frequent menstrual bleeding (polymenorrhea), and/or irregular menstrual cycles. Several contributing factors have been explored, including stress,^16^ BMI,^17^ and age at menarche,^18^ and as discussed above, the precise mechanisms and consequences resulting from these individual differences could also contribute to the expression of ADHD symptoms.^20–22^

Other individuals may be diagnosed with a menstrual disorder. One key example is Polycystic Ovary Syndrome (PCOS). PCOS is a chronic endocrine disorder with a complex presentation, with symptoms involving menstrual difficulties, infertility, metabolic issues, acne, and excess hair growth in a male-pattern distribution, among other symptoms.^23^ It is the most prevalent endocrine disorder affecting menstruators in their reproductive years,^23,24^ with 4-10% of menstruators globally having PCOS.^23^ On the hormonal level, PCOS is characterized by hyperandrogenism, in which individuals produce excessive levels of androgens.^25^

Beyond individual differences in menstruation itself, there is also a relatively broad body of literature exploring physical and mood-related changes in the days preceding menstruation, during the luteal phase. The fluctuations in sex hormones during this time are robustly linked to premenstrual symptoms, which affect 47.8% of menstruating individuals.^26^ These manifestations range from psychological effects, such as irritability, tearfulness, anxiety, and depressed mood,^26^ to physical symptoms, including abdominal bloating, headaches, and breast tenderness.^26^ Like ADHD symptoms, although these experiences occur in nearly half of menstruators, for some they become clinically significant and are diagnosed as Premenstrual Dysphoric Disorder (PMDD). This disorder is characterized by severe physical, behavioural, and affective symptoms during the luteal phase of the menstrual cycle,^27^ and affects an estimated 1.6% of menstruators worldwide.^28^

Studies that currently explore the impact of the luteal phase in the context of ADHD have primarily focused on mood-related symptoms in individuals with ADHD,^29,30^ with reported increases in anxiety and depression during this phase.^30^ However, little attention has been given to understanding fluctuations in core ADHD symptoms during this period or across the menstrual cycle more broadly. Only recently has the relevance of menstrual cycle dynamics for ADHD symptom presentation and fluctuation begun to receive focused attention.

### Existing reviews and theoretical perspectives

A systematic review has examined sex hormones and ADHD symptoms across the female lifespan, including puberty, menstrual cycles, and pregnancy.^31^ In addition, a narrative review has explored the effectiveness of psychostimulant medication throughout the menstrual cycle.^32^ A recent article has also presented a novel theoretical framework proposing that fluctuations in ovarian hormones may actively influence ADHD symptoms, with particular vulnerability during sharp drops in estrogens.^33^ Specifically, Eng et al. (2024) propose that estradiol supports dopaminergic signalling in prefrontal circuits involved in executive control. Rapid decreases in estrogen may therefore temporarily disrupt these processes and contribute to ADHD symptoms such as inattention and impulsivity.^33^ To our knowledge, no scoping review has mapped menstrual health characteristics (e.g., cycle phase, menstrual pain, bleeding patterns, comorbid conditions) in relation to ADHD symptom presentation among individuals presenting ADHD symptoms with or without an ADHD diagnosis. Addressing this gap is essential for clarifying sex-specific ADHD trajectories and improving the accuracy of diagnosis and treatment for menstruating individuals.

## Review objectives

### Primary research questions

1. What is known about the relationship between menstruation-related characteristics (e.g., menstrual pain, heavy bleeding, irregular cycles, and cycle-related symptom changes) and ADHD symptom presentation in menstruating individuals AFAB?
2. What is the methodological quality of the existing literature, with respect to sample selection and justification, ascertainment of ADHD and menstrual characteristics, control for confounders, outcome assessment, and statistical analysis?

## Methods

This scoping review was conducted in consultation with the JBI methodology (including use of the JBI Protocol Template)^34^ and PRISMA-ScR Checklist^35^ for scoping reviews. The completed PRISMA-ScR checklist is provided in Supplementary File 1.

### Protocol and registration

No review protocol was preregistered or published prior to conducting this review.

### Inclusion and exclusion criteria

#### Participants

Considering the underdiagnosis of individuals AFAB with neurodevelopmental disorders, particularly ADHD,^36,37^ this review aimed to include ADHD symptoms in menstruators as broadly as possible. Studies were included if they involved participants with a diagnosis of ADHD, reported ADHD symptoms, or menstrual health-related differences in inattention and/or hyperactivity-impulsivity.

#### Concept

This review focused on the relationship between menstruation-related characteristics (e.g., heavy, painful, or irregular cycles, hormonal fluctuations, and cycle-related symptom changes) and the presentation of ADHD symptoms. The concept of interest included how menstrual health factors may influence the onset, severity, maintenance, or fluctuation of ADHD symptoms such as inattention, hyperactivity, and/or impulsivity.

#### Context

To capture the widest possible view of the literature on ADHD and menstrual health, no geographical restrictions were applied. For each included study, we noted whether cultural or geographical factors were discussed. French and English studies were included in the search.

#### Types of sources

This scoping review considered all peer-reviewed, published primary research study designs, including experimental and quasi-experimental designs (e.g., randomized and non-randomized controlled trials, before-and-after studies), analytical observational studies (e.g., cohort, case-control, and cross-sectional designs), descriptive studies (e.g., case series and individual case reports), and qualitative studies that used systematic and replicable approaches (e.g., qualitative description or thematic analysis). Systematic reviews, meta-analyses, graduate theses, and conference abstracts were excluded.

All eligibility criteria are outlined in Appendix I.

### Search strategy

A structured keyword-based search strategy was developed in consultation with the Subject Librarian for the Department of Psychology (Mr. Dominic Desaulniers) at the University of Montreal to identify relevant literature for this scoping review. An initial list of keywords was created based on the review objectives, with an emphasis on ADHD, menstruation, and menstrual phase and health features.

The finalized search strategy was implemented across three electronic databases: MEDLINE (via Ovid), PsycINFO (via Ovid), and Web of Science on the 18^th^ of July 2025. A keyword-based search strategy using Boolean operators and truncation was applied across all three databases without modification for database-specific indexing systems or controlled vocabulary (e.g., MeSH or APA Thesaurus terms were not applied).

The reference lists of related review articles retrieved during the search were also manually screened to identify additional eligible sources. No filters for study design, language, or publication date were applied at the initial stage. Grey literature, preprints, and unpublished theses were included in the initial search, though unpublished graduate theses, conference abstracts, and articles in non-peer reviewed journals were later screened out. The full search strategy is provided in Appendix II.

### Study selection

691 studies were identified in the search and imported to Covidence for screening.^38^ 210 duplicates were identified and removed by Covidence, and 50 duplicates were removed manually, leaving 431 titles and abstracts to be screened.

Titles and abstracts were screened by two independent reviewers (1. G.K., 2. M.B.G.) against the inclusion and exclusion criteria for the review. The full eligibility criteria for the review are available in Appendix II. Any disagreements that arose between the reviewers at each stage of the screening process were resolved through discussion, or with an additional reviewer (S.L.). Of the 431 studies, 409 were deemed irrelevant, and 22 were retained for full-text review.

**Figure 2.**
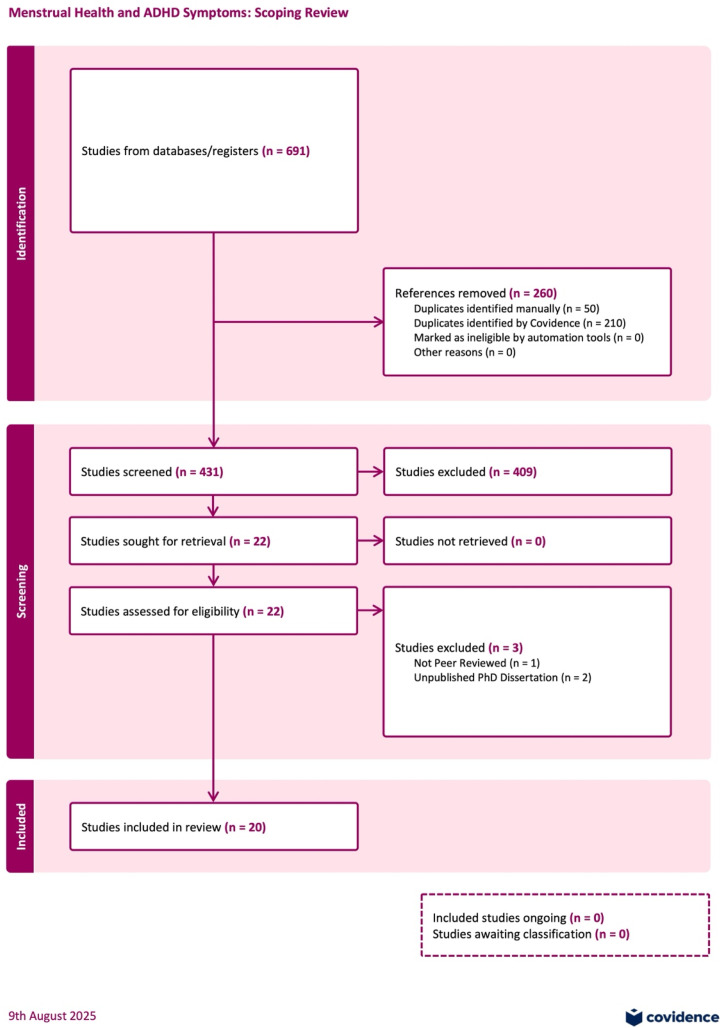
PRISMA flow diagram: Menstrual health and ADHD symptoms: Scoping review. *Note.* PRISMA-ScR flow diagram depicting the study selection process for the current scoping review. A total of 691 records were identified through PsycINFO (Ovid), MEDLINE (Ovid), and Web of Science. After removal of 260 duplicates (210 by Covidence and 50 manually), 431 records were retained for title and abstract screening. Of these, 409 were excluded based on the predefined inclusion and exclusion criteria, leaving 22 records for full-text review. Three full-text articles were excluded: two unpublished PhD dissertations and one article published in a non-peer-reviewed popular science journal. 20 studies met the eligibility criteria and were included in the final synthesis.

The full texts of 22 studies were assessed against the inclusion and exclusion criteria by the two independent reviewers (G.K., M.B.G.). Reasons for exclusion of full-text sources that did not meet inclusion criteria were recorded and are reported in the results section (Figure 2). 19 articles were retained for data extraction and analyses. It is important to note that one article^39^ was made up of two studies. As a result, 20 studies made up the final review of the literature.

### Data extraction

Data from the final set of studies (n = 20) was extracted by both reviewers using an adapted version of Covidence’s Data Extraction Template. The modifications made to Covidence’s template included the addition of questions on ADHD symptom and menstrual health assessment, sex and gender distribution, and reviewer-identified themes.

Extracted data included study characteristics (author, year, country, study design), participant characteristics (sample size, age, sex/gender), the concept(s) investigated, the context (setting, population), study methods, key findings and conclusions relevant to the review questions, and reviewer-identified themes as a preliminary step for later thematic grouping. The Covidence Extraction Template is provided in Appendix III. The data extraction tool was not modified or revised during the process of data extraction. As previously, any disagreements were resolved through discussion, with the third reviewer (S.L.) consulted as needed.

### Quality assessment

A critical appraisal of individual sources of evidence (i.e., quality assessment) was conducted, although this step is not required for scoping reviews according to JBI guidance. The appraisal was conducted to provide additional context regarding the methodological rigor of the included studies.

A Quality Assessment Form was adapted from Osianlis et al. (2025), whose systematic review on ADHD and sex hormones in females employed a lifespan approach across hormonal transitions. Given our more specific focus on menstruation and its features, we adapted their Risk of Bias criteria, which was based on the Newcastle-Ottawa Scale (NOS),^40^ to fit the design of the present review. The categories in this adapted form assessed selection (representativeness, sample size justification, inclusion/exclusion criteria, ascertainment of ADHD and menstrual characteristics) (5 stars), comparability (control of confounders) (2 stars), and outcome (measurement validity and appropriateness of statistical analyses) (3 stars), with total scores ranging from 1 to 10 stars. The adapted form, with modifications highlighted, is provided in Appendix IV.

### Data analysis and presentation

Data from the included studies were charted in Covidence and exported to Zotero for synthesis. Guided by the review objectives, studies were grouped according to six key themes that emerged during the data extraction process, and through reviewer discussion. A narrative synthesis was used to describe how the results addressed the review questions. No quantitative meta-analysis was conducted, consistent with JBI scoping review methodology.^34^

## Results

### Search results

### Inter-rater reliability

Inter-reliability for title and abstract screening was high, with a proportionate agreement of 96.8% and a Cohen’s kappa of 0.69, indicating substantial agreement.^41^ Inter-rater reliability for full-text screening was excellent, with 100% agreement and a Cohen’s kappa of 1.0, indicating perfect agreement beyond chance.^41^ 74.6% of full-text articles were agreed upon for inclusion, with 3 articles (25.4%) being excluded.

### Study characteristics

Once again, it is important to note that one article^39^ was made up of two studies. As a result, both studies were assessed separately in the Study Characteristics and Quality Assessment. As shown in Supplementary Table 1, most studies were conducted in Turkey (n = 5, 26.31%). 4 studies (21.05%) were conducted in Taiwan, 3 in China (15.79%), and 2 in the Netherlands (10.53%). Ghana, Sweden, the United Kingdom, Canada, Australia, and the United States each produced one study (5.26% each). The literature spanned 2014 to 2025, with most (70%, n = 14) articles having been published during or after 2020.

The majority (42.11%, n = 8) of studies employed a non-randomized experimental design. 21.05% (n = 4) of the studies were cross-sectional, 15.79% (n = 3) were qualitative, and 15.79% (n = 3) were case reports. One study was a case series, and one was longitudinal (5.3% each). Most studies were set in the community (57.89%, n = 11), with 36.84% (n = 7) having been conducted in clinical settings, and 10.53% (n = 2) having been conducted in educational settings. Sample sizes ranged from 1 to 405, with most studies containing under 200 participants (80%, n = 16).

Availability of age data varied from one study to another. 15 (75%) studies reported age ranges, with ages ranging from 13-49 across all studies reporting this information. 15 studies reported age means, with the mean age across all studies being 23.58 years (SD = 6.3, median = 22.79). 65% (n = 13) of the studies incorporated multiple assessment time points. All study participants were assigned female at birth, and only 2 studies report information regarding participants’ gender identity. 45% (n = 9) of studies explored ADHD symptoms regardless of a formal diagnosis, 40% (n = 8) of studies included individuals with formal ADHD and/or other diagnoses (e.g. PMDD, PCOS), and 15% (n = 3) of studies explored menstrual-pain-related inattention and impulsivity outside of the context of ADHD. A comprehensive description of the types of measures used is available in Appendix V. A summary of all study designs and assessment timepoints is available in Appendix VI.

### Quality assessment

The included studies were ranked based on the following Quality Assessment Form score ranges: *0 to 4 = Unsatisfactory, 5 to 6 = Satisfactory, 7 to 8 = Good, 9 to 10 = Very Good*. Half of the included studies were deemed *Very Good* (n = 10, 50%). 3 studies (15%) were deemed *Good*, 3 (15%) were deemed *Satisfactory*, and 4 (20%) were deemed *Unsatisfactory*. The studies are presented thematically, with their Quality Assessment scores considered alongside their findings. Quality assessment results are also summarized in Appendix V.

### Thematic synthesis of findings

Themes were generated during the data extraction process and finalized through consensus among the authors. They are mapped below in order of prevalence across the included studies, from most to least frequent. It is important to note that, though we use the term “individuals AFAB” throughout this article, we used the terms employed by the authors in discussing their results (e.g., women, females).

### Comorbid ADHD symptoms in individuals with menstruation-related conditions

The most prominent theme across the literature (n = 5) was the co-occurrence of ADHD symptoms among individuals with menstruation-related conditions, particularly Premenstrual Dysphoric Disorder (PMDD) and Polycystic Ovary Syndrome (PCOS). Across studies, participants with these conditions often reported greater inattention and impulsivity difficulties compared to those without. However, the nature of these associations differed by condition.

#### Premenstrual dysphoric disorder

Four studies^29,42–44^ examined ADHD symptoms among individuals diagnosed with PMDD. As previously mentioned, PMDD is characterized by severe physical, behavioural, and affective symptoms occurring pre-menstrually.^27^ Notably, all of these studies^29,42–44^ appear to have originated from the same Taiwanese research group and demonstrated consistent findings linking PMDD with heightened inattention, among other symptoms, throughout the menstrual cycle. Although the authors examined associations between PMDD, ADHD, and several other domains of mental health, cognition, and everyday functioning, only results relevant to ADHD are discussed below.

Lin et al. (2021) found that women with PMDD reported significantly higher levels of inattention compared to control participants. Lin et al. (2022) expanded on these findings by comparing outcomes across the early- and late-luteal phases of the menstrual cycle. In this study, women with PMDD showed higher levels of inattention in both the early- and late-luteal phases relative to controls, with inattention emerging as the variable most strongly associated with PMDD in the late-luteal phase.^44^ In both studies, women were considered part of the PMDD group if they met the *Diagnostic and Statistical Manual of Mental Disorders, Fifth Edition* (DSM-5) criteria for PMDD.^45^

Lin et al. (2024) investigated the prevalence of comorbid ADHD diagnoses and symptoms among women with PMDD, focusing on inattention throughout different phases of the menstrual cycle. In this study, adult ADHD and PMDD diagnoses were confirmed by a psychiatrist.^29^ Women with PMDD were significantly more likely than controls to have an ADHD diagnosis (27.6% of the PMDD group). Across menstrual phases, participants with PMDD demonstrated greater difficulty sustaining attention compared to controls, with the steepest decline observed from the pre-ovulatory to late-luteal phase.^29^ Among participants with comorbid ADHD, inattention scores were elevated across phases but did not significantly differ from those with PMDD alone, suggesting that PMDD itself may contribute substantially to attentional difficulties, independent of an ADHD diagnosis.^29^

Ko et al. (2024) examined hormonal and neurotrophic correlates of PMDD symptoms across the pre-ovulatory, mid-luteal, and late-luteal phases. PMDD diagnoses were confirmed using the DSM-5 criteria for PMDD.^45^ No significant differences were observed in estrogen or progesterone levels across phases. Women with PMDD reported higher inattention scores compared to controls during all three phases compared to control participants.^42^ Within the PMDD group, lower levels of brain-derived neurotrophic factor (BDNF) were associated with greater inattention, suggesting that reduced neurotrophic activity may contribute to attentional difficulties.^42^ Additionally, among all participants, vascular endothelial growth factor (VEGF) levels were negatively correlated with inattention, further implicating neurobiological mechanisms in the attentional symptoms observed among individuals with PMDD.^42^

All four studies received a “Very Good” quality rating (9/10), suggesting that the evidence base, while limited in geographic scope, is methodologically robust. One point was deducted from all studies due to the lack of a power analysis and/or sample size justification.

Across all studies, individuals with PMDD consistently exhibited greater inattention than controls, with symptom severity tending to increase from the pre-ovulatory to late-luteal phase.^29,42–44^ Collectively, these findings suggest that PMDD is associated with heightened attentional vulnerability that may fluctuate with hormonal and neurobiological changes across the menstrual cycle.

#### Polycystic ovary syndrome

One article^24^ examined ADHD symptoms in women with PCOS. As previously mentioned, PCOS is characterized by hyperandrogenism.^25^ Because androgens influence neural systems involved in reward processing, inhibition, and emotional regulation, higher androgen exposure has been hypothesized as a potential contributor to ADHD symptom development, though these hypotheses have historically targeted ADHD development in individuals assigned male at birth (AMAB).^46^

Hergüner et al. (2015) compared ADHD symptoms in women with and without PCOS. Though it does not explicitly explore these symptoms in relation to the menstrual cycle, its inclusion felt important as PCOS is widely experienced among menstruators. Their findings report that women with PCOS showed significantly higher total adult (via *Adult ADHD Self-Report Scale*) and total childhood (via *Wender Utah Rating Scale for the Attention-Deficit/Hyperactivity Disorder*) ADHD scores than controls, with particularly elevated hyperactivity-impulsivity subscale scores.^24^ However, there were no significant group differences in inattention scores on either measure.^24^

This study received a “Very Good” quality rating (9/10), with one point deducted for the absence of a power analysis and/or sample size justification. Overall, these findings suggests that the association between PCOS and ADHD may be more closely tied to impulsivity than to attentional difficulties, potentially reflecting the neurobehavioral effects of androgen excess.^46^ PCOS diagnoses were confirmed using the *Rotterdam Criteria*.^24^

### Dysmenorrhea, menorrhagia, irregular menstruation and ADHD symptoms

The second theme (n = 4) that emerged explored how menstrual difficulties, including severe menstrual pain (dysmenorrhea), heavy menstrual bleeding (menorrhagia), and irregular menstruation were linked to greater ADHD symptom severity, functional disruption, and higher comorbidity rates.^30,47–49^

#### Dysmenorrhea

Kabukçu et al. (2021) examined the relationship between dysmenorrhea and various psychological symptoms and aspects of everyday life. Their results related to ADHD are presented here. They found that adolescents with severe dysmenorrhea reported significantly higher inattention and hyperactivity-impulsivity scores, suggesting that menstrual pain interfering with daily activities may be linked to greater ADHD symptom burden.^47^ This study received a “Good” quality rating (8/10), losing points for lack of power analysis/sample size justification and unclear description of inclusion/exclusion criteria.

Lockinger and Gagnon (2023) explored the relationship between ADHD, dysmenorrhea, emotional regulation, and psychological well-being in females with a self-reported ADHD diagnosis. Their results pertaining to ADHD and dysmenorrhea are presented here. They observed a moderate positive correlation between ADHD symptom severity and dysmenorrhea severity in adult women, with a 95% comorbidity rate.^30^ This result emphasizes the potential importance of considering the co-occurrence of ADHD symptom severity and dysmenorrhea in clinical and research contexts. This study received a “Very Good” quality rating (10/10), with no observed methodological limitations.

#### Menorrhagia

MacLean et al. (2025) examined the relationship between iron deficiency, heavy menstrual bleeding, and ADHD symptoms. They reported that women with ADHD symptoms were more likely to experience heavy menstrual bleeding and symptoms of iron deficiency,^48^ further supporting a relationship between ADHD and menstrual health difficulties. This study received a “Very Good” quality rating (10/10), with no observed methodological limitations.

#### Irregular menstruation

Yuan et al. (2024) examined the relationship between menstrual and mental health longitudinally in school-aged girls. Multiple areas of mental health (PTSD, depression, anxiety, ADHD, insomnia, psychotic-like experiences, non-suicidal self-injury, and suicidal ideation, plan, and attempt) were assessed. Only their results pertaining to ADHD are discussed here. The authors found that menstrual irregularity, but not early menarche or menstrual pain, was associated with a threefold increase in the likelihood of persistent ADHD symptoms over one year.^49^ This suggests that irregular cycles may contribute to the maintenance rather than the onset of attentional difficulties. This study received a “Good” quality rating (8/10), losing points for lacking a power analysis/sample size justification, and for providing an unclear description of inclusion and/or exclusion criteria.

Collectively, these findings suggest that menstrual pain, bleeding abnormalities, and cycle irregularity may exacerbate or co-occur with ADHD symptoms across different timepoints in the development and maintenance of disorder symptoms. None of the above studies explored hormonal mechanisms underlying these associations, and causal mechanisms linking these menstrual features to each other and/or ADHD remain unclear.

### Fluctuations in ADHD symptoms throughout the menstrual cycle

The third theme (n = 4) explored premenstrual intensification of ADHD symptoms,^39,50,51^ with some qualitative evidence of reduced psychostimulant medication effectiveness during this period.^50^

Bürger et al. (2024) qualitatively explored how individuals with a self-reported ADHD diagnosis experienced symptom changes across the menstrual cycle. Although participants described a range of emotional and executive functioning difficulties (e.g., irritability, anxiety, and task initiation problems), only findings related to attention are discussed here. Participants consistently qualitatively reported worsening ADHD symptoms during the mid-luteal phase and menstruation, with specific difficulties in sustaining concentration, staying organized, and managing forgetfulness.^50^ Several participants also noted that their ADHD medication felt less effective during the mid-luteal phase or that its effectiveness fluctuated across their cycle overall.^50^ These accounts suggest that perceived attentional regulation and treatment response may qualitatively vary across menstrual phases among individuals with ADHD.^50^ This study received a “Very Good” quality rating (9/10), losing points for providing an unclear description of inclusion and/or exclusion criteria.

Roberts et al. (2018) examined how reproductive steroid hormones relate to ADHD symptoms across the menstrual cycle in a community sample. The authors assessed within-person fluctuations in inattention and hyperactivity-impulsivity in relation to estradiol, progesterone, and testosterone, while accounting for impulsivity-related traits such as Sensation Seeking and Urgency.^51^ Higher average estradiol and progesterone levels were associated with lower inattention, and greater variability in progesterone across the cycle predicted fewer inattentive symptoms.^51^ That is, women whose progesterone levels fluctuated more from day to day tended to report fewer attention difficulties, suggesting that more dynamic progesterone changes, rather than consistently low or high levels, may be linked to better attentional functioning. However, these effects differed by impulsivity traits: among participants high in Sensation Seeking or Urgency, interactions between estradiol and progesterone predicted both inattention and hyperactivity-impulsivity, such that lower estradiol during elevated progesterone was linked to greater symptom expression among those high in impulsivity traits.^51^ For those high in Sensation Seeking, elevated testosterone amplified hyperactivity-impulsivity, and estradiol interacted with testosterone, such that higher estradiol reduced inattention when testosterone was elevated but increased it when testosterone was low.^51^ Within-person analyses showed that inattentive symptoms peaked during the post-ovulatory phase, particularly among participants high in impulsivity traits.^51^ Overall, these findings suggest that ADHD symptoms may vary across the menstrual cycle in a state-like manner, shaped by interactions between hormone levels and individual impulsivity profiles. This study received a “Very Good” quality rating (10/10), with no observed methodological limitations.

Zhuang et al. (2020) explored how cognitive control over impulsive behaviour fluctuated across the menstrual cycle when measured via fMRI in a community sample. As previously mentioned, this article consisted of two studies. As a result, the studies were assessed separately in the Quality Assessment and their results synthesized separately. In Study A, women showed greater ability to delay rewards, indicating better impulse control, during the mid-luteal phase compared to the late follicular phase.^39^ This suggests that hormonal changes in the luteal phase may enhance cognitive regulation of impulsivity. During the mid-luteal phase, women showed increased activation of the dorsolateral prefrontal cortex (dlPFC) and stronger functional connectivity between the dlPFC and the dorsal striatum (DS) compared to the late follicular phase.^39^ In this way, during the mid-luteal phase, elevated dlPFC engagement may reflect stronger top-down modulation of striatal reward circuitry (via dlPFC-DS connectivity), aligning with behavioural evidence for enhanced impulse control.^39^ This study received a “Satisfactory” quality rating (6/10), losing points for lacking a power analysis/sample size justification, providing no description of an ADHD-specific ascertainment of symptoms, and for using an unvalidated measure of ADHD (fMRI measures are described but not validated measures of ADHD/impulsivity).

In study B, Zhuang et al. (2020) investigated resting state fMRI activity and its associations with hormonal levels and self-reported impulsivity across the menstrual cycle. Impulsiveness was measured using the *Barrett-Impulsiveness Scale* (BIS-11),^52^ specifically measuring attentional impulsiveness, motor impulsiveness, and non-planning impulsiveness. Results showed greater right dlPFC activation in the mid-luteal phase compared to the late follicular phase.^39^ Further, the magnitude of functional connectivity in the right dlPFC was negatively correlated with BIS-11 attentional impulsivity.^39^ Saliva assays indicated that estradiol levels were positively correlated with dlPFC activity during the mid-luteal phase, suggesting that the augmented cognitive control seen in this phase may be accounted for by hormone levels.^39^ These findings built on Study A by extending the measure of impulsivity beyond a behavioural task-based measure to include trait-level, self-reported measures of impulsivity. This study received an ‘Very Good’ quality rating (9/10), with points only lost for lacking a power analysis/sample size justification. Though the study did not directly examine ADHD, its use of a validated measure of an ADHD-related symptom (impulsivity) makes it a valuable contribution to the review by providing insight into the neural and hormonal mechanisms underlying impulsive behaviour throughout the menstrual cycle.

Across these studies, patterns of ADHD-related symptoms varied across the menstrual cycle, with some evidence suggesting that attentional regulation may fluctuate in relation to changing estrogen levels.^15^ Although several findings point to increased difficulties during the mid-luteal phase and menstruation, when estrogen is lowest,^15^ other results indicate higher cognitive control and reduced impulsivity during the mid-luteal phase.^39^ Therefore, behavioural traits, hormonal profiles, and menstrual cycle phase appear to interact in complex ways to influence how ADHD-related symptoms may vary across the menstrual cycle.

### Osmotic-Release Oral System Methylphenidate (OROS-MPH) and menstrual side-effects

The fourth theme (n = 3) explored how Osmotic-Release Oral System Methylphenidate (OROS-MPH), also known as Concerta, was associated with heavy menstrual bleeding, cycle length changes, and menorrhagia, which typically resolved after discontinuation or dose adjustment.^53–55^ This theme emerged exclusively from case studies. As will be discussed in the following section, these findings should be interpreted with caution given the very small sample sizes and absence of experimental control.

Coskun & Adak (2017) reported a case of excessive and frequent menstrual bleeding (hypermenorrhea and polymenorrhea) in an adolescent girl with ADHD prescribed OROS-MPH. These menstrual abnormalities resolved following the prescription of an oral contraceptive pill. After five months of OROS-MPH treatment, she discontinued this medication as well as her contraceptive pill, and reported that her menstrual cycle remained regular following this.^53^ Mutlu et al. (2016) observed increased menstrual cycle length following the use of OROS-MPH in an adolescent girl with diagnosed ADHD, which ceased following a reduction of the OROS-MPH dose. Finally, Ozdag et al. (2022) documented OROS-MPH-induced menorrhagia in twin adolescent girls, with symptoms resolving after stopping the medication. Collectively, these reports suggest a possible link between OROS-MPH use and menstrual irregularities in adolescents, though mechanisms remain unclear and evidence is very limited.

All three studies received “Unsatisfactory” quality ratings (1/10, 2/10 and 2/10, respectively), due to the lack of representativeness of their sample, lack of justification of their sample size, lack of inclusion/exclusion criteria, lack of comparability, lack of methodological validation and statistical control. As previously mentioned, though collectively these findings suggest a potential link between OROS-MPH use and menstrual irregularities, they should be interpreted with caution due to their methodological limitations.

### Menstrual pain-related attentional interference

The fifth theme (n = 2) examined how menstrual pain impaired attention during pain phases compared to pain-free phases.^56,57^ These studies did not explicitly aim to investigate ADHD but instead reported attentional difficulties related to menstrual pain. We chose to include them nonetheless, as they provide important context for understanding how inattention may relate to the menstrual cycle and the role that pain may play in modulating attention.

Aziato et al. (2014) qualitatively explored the experiences of dysmenorrhea in students. Multiple themes emerged, and the theme that was of interest to this study and that will be discussed was “absenteeism and inattentiveness”.^56^ That is, certain participants reported an inability to concentrate in classes during their menses, often missing school due to menstrual pain 56. These symptoms and their consequences were also reported to disrupt daily activities, impairing their concentration.^56^ The authors concluded that severe dysmenorrhea had an important negative impact on female students’ academic and personal lives.^56^ This study received a “Satisfactory” quality rating (6/10), losing points for limited sample representativeness, unclear ADHD symptom ascertainment, and the use of a non-validated method for measuring ADHD symptoms. However, its qualitative interview approach was well-described and aligns with gold-standard practices for qualitative research.

Keogh et al. (2014) investigated how menstruation-related pain affected attention across multiple cognitive tasks. Each task captured different components of attention potentially relevant to ADHD, including selective attention (flanker task), attention span (n-back task), attentional switching (switching task), and divided attention (dual task).^57^

Overall, participants performed worse during the menstrual pain phase compared to the pain-free phase, with slower reaction times and lower accuracy across all tasks.^57^ Specifically, menstrual pain was linked to slower responses on the flanker task, more false alarms on the n-back task, reduced accuracy on the switching task, and poorer performance on the dual-task condition.^57^ These findings suggest that menstrual pain broadly interferes with attention, leading to general performance dampening rather than task-specific deficits. This study received a “Satisfactory” quality rating (6/10), losing points for lack of power analyses/sample size justification, unclear ADHD symptom ascertainment, and the use of a non-validated method for measuring ADHD symptoms, though their methods were clearly described and available in the manuscript.

Though study designs and goals differed substantially in this theme, taken together, these studies suggest that menstrual pain was qualitatively and quantitatively related to difficulties in sustaining attention.^56,57^

### Menstrual cycle-tailored interventions for individuals with ADHD and menstrual symptom variation

The sixth and final theme that emerged (n = 2) discussed menstrual-cycle informed interventions for ADHD symptom management. The two studies in this theme,^58,59^ which appear to be from one group in the Netherlands, explore psychopharmacological^58^ and therapeutic^59^ interventions tailored to individuals with ADHD who experience cycle-related worsening of symptoms.

De Jong et al. (2023) presented a case-series of nine women with ADHD and premenstrual symptom worsening whose psychostimulant doses were increased during the premenstrual (luteal) phase. Across 6-24 months of follow-up, all participants self-reported reduced inattention, irritability, and energy dips, with increased symptom stability across the cycle and minimal side effects.^58^ Participants reported that their dosage adjustment appeared to counteract the luteal-phase reduction in stimulant efficacy.^58^ It is important to keep in mind that this is a case-series and was attributed an “Unsatisfactory” quality rating (3/10). This is due to the lack of representativeness of its sample, its lack of power analyses/sample size justification, unclear description of inclusion/exclusion criteria, inadequate degree of statistical control and lack of description of statistical analyses. As a result, these results should be interpreted with caution.

De Jong et al. (2024) qualitatively analyzed experiences from a novel, menstrual cycle-informed ADHD group intervention combining psychoeducation about the menstrual cycle with tracking tools and discussions of ADHD-related challenges. Results indicated that participants described the group as a safe and validating environment that increased self-awareness of cyclical patterns, self-acceptance, and communication about treatment needs.^59^ Specifically, participants identified six themes: *safety-sharing-welcome, recognition, good fit, eye-opener, take(n) seriously,* and *empathy*, which reflected two overarching experiences of *understanding* and *connection* within the group.^59^ They reported greater recognition of how hormonal fluctuations affected their focus, motivation, and self-regulation as a result of participation.^59^ This study received a “Good” quality rating (7/10). Points were deducted due to lack of sample representativeness, power analyses/sample size justification, and qualitative analysis description in the methodology.

Together, these studies illustrate preliminary evidence that menstrual-tailored ADHD interventions, both pharmacological and psychoeducational, may have the future potential to enhance symptom management and treatment satisfaction for individuals experiencing menstrual cycle-related fluctuations in their ADHD symptoms.

## Discussion

### Key insights from the current literature

The current literature on ADHD symptoms and menstrual health, though limited in quantity (n = 20), is remarkably diverse in its methods, study designs, objectives, and geographical contexts. Through the present scoping review, the following six themes emerged^1^: comorbid ADHD symptoms in individuals with menstruation-related conditions^2^; dysmenorrhea, menorrhagia, irregular menstruation and ADHD symptoms^3^; fluctuations in ADHD symptoms throughout the menstrual cycle^4^; OROS-MPH and menstrual side-effects^5^; menstrual pain-related attentional interference; and menstruation-tailored interventions for individuals with ADHD and menstrual symptom variation.

Across all themes, it became clear that menstrual health features are related to ADHD symptomatology. Firstly, there appears to be a growing link being established between menstruation-related disorders and ADHD. For example, multiple studies linked PMDD with inattention,^29,42–44^ with one study linking low levels of BDNF with greater inattention in the context of PMDD.^42^

BDNF is a neurotrophin that is heavily implicated in various cognitive and affective processes.^60^ However, the literature examining BDNF, sex differences, and ADHD, particularly in clinical populations, remains extremely limited. A study exploring the role of BDNF in ADHD symptoms among school-aged girls and boys found that BDNF levels were higher in boys with ADHD compared to control boys.^61^ An opposite pattern was observed among girls in the same study.^61^ Specifically, girls with ADHD showed lower BDNF levels relative to control girls.^61^ Girls with lower BDNF levels also committed more omission errors on the Conners’ Continuous Performance Test,^61^ a measure commonly interpreted as reflecting inattention.^62^ These findings suggest that there may be sex-specific expressions of BDNF in children with symptoms of ADHD.

Reduced levels of BDNF have also been associated with depressive symptoms.^60^ In the context of our review findings, the association between lower BDNF levels, depressive symptoms, and ADHD symptoms in females may explain the cyclical changes in attentional functioning observed in menstruators with PMDD.^42^ However, causal links between BDNF expression, PMDD, and ADHD symptoms remain to be established.

One study also linked PCOS to impulsivity.^24^ As previously mentioned, PCOS is characterized by the hyper-expression of androgens, like testosterone.^25^ There exists a relatively broad body of literature examining androgen exposure and ADHD risk, but it has largely focused on populations AMAB due to the role of testosterone in this proposed mechanism.^46^ Within this literature, exposure to androgens from early pregnancy through adolescence has been linked to ADHD symptoms, particularly externalizing symptoms such as impulsivity in youth.^63,64^ Specifically, this association has been observed in relation to genetic variation in CAG and GGC polymorphisms in the androgen receptor (AR) gene, which are associated with increased androgen activity.^63^

This genetic model provides an interesting foundation from which to interpret our review’s findings on PCOS. CAG polymorphisms have also been observed in individuals diagnosed with PCOS.^65^ It is therefore possible that, similarly to studies examining AR gene variation in youth with ADHD, potential genetic differences in individuals with PCOS linked to hyperandrogenism may also contribute to the increased presence of ADHD symptoms in this population, particularly impulsivity. Most importantly, this underscores the need to include individuals AFAB in this literature, as developmental androgen exposure may also affect them, with implications for both neurodevelopment and reproductive health.

Together, these findings suggest that distinct, sex-specific hormonal dynamics, including hyperandrogenism in PCOS and cycle-linked BDNF changes in PMDD, may differentially shape ADHD symptom expression in menstruating individuals with diagnoses of menstruation-related disorders.

Secondly, beyond clinical diagnoses, different menstrual health difficulties appear to have distinct relationships to ADHD symptoms in the general population. For example, two studies bi-directionally linked dysmenorrhea with both inattentive and hyperactive-impulsive profiles of ADHD, with high comorbidity rates between painful menstruation and ADHD symptom severity.^30,47^ Even in studies not explicitly studying ADHD, menstrual pain was associated with attentional issues in qualitative and quantitative settings.^56,57^ One study^48^ found a relationship between ADHD symptoms, heavy menstrual bleeding (menorrhagia), and iron deficiency. Heavy menstrual bleeding has been robustly linked as a primary contributor to iron deficiency and, in severe cases, iron deficiency anemia.^13^ Iron deficiency has also been identified as a factor associated with ADHD in children.^66^ Despite results from adult studies linking iron deficiency with ADHD symptoms being limited and mixed,^67^ one possible pathway may be that recurrent heavy menstrual bleeding can lower iron stores,^13^ with iron being needed for oxidative metabolism, myelination during development, and neurotransmitter synthesis in the brain 68. Iron is also a cofactor for tyrosine hydroxylase, the rate-limiting enzyme in dopamine synthesis, with reduced iron availability potentially disrupting dopamine production and related signalling.^68,69^ In animal models, iron deficiency reduces dopamine transporter density and striatal dopamine uptake, increases extracellular dopamine, and alters dopamine-related gene expression in the ventral midbrain, suggesting that low iron has the potential to disrupt dopamine regulation at multiple levels.^70,71^ In this way, it may be the case that heavy menstrual bleeding contributes to ADHD symptoms in some individuals through iron-related changes in dopamine regulation. Consistent with this, better iron status in nonanemic young females has been associated with better attention and executive functioning.^72^ Here, Maclean et al.’s (2025) findings highlight the importance of considering menstruation as a potential contributor to iron deficiency in pathways linking iron status and ADHD symptoms in adults. Taken together, the present reviews findings on menstrual pain and heavy menstrual bleeding suggest that specific menstrual features may have broad impacts both within and beyond clinical ADHD populations.

Though their methodological limitations are important to consider, some case studies found that certain psychopharmacological ADHD treatments were linked to the development of menstrual pathologies,^53–55^ which ceased after dose adjustment or medication discontinuation. Coskun and Adak (2017) hypothesized that the menstrual abnormalities observed in their 17-year-old patient treated with OROS-MPH were due to hormonal rather than hematologic factors, given their resolution with oral contraceptive use. However, they did not propose a specific mechanism to explain this effect. In contrast, Mutlu et al. (2016) described a 17-year-old girl whose menstrual cycle lengthened following OROS-MPH treatment and normalized once the dosage was reduced. They suggested that methylphenidate’s dopaminergic and noradrenergic effects may disrupt the normal pulsatile release of gonadotropin-releasing hormone (GnRH) and luteinizing hormone (LH), potentially through alterations in ghrelin and leptin signalling, which in turn interferes with neuroendocrine regulation and lengthens the menstrual cycle.^54^ Similarly, Özdag et al. (2022) proposed that methylphenidate’s dopaminergic activity may disrupt GnRH and LH secretion or alter platelet function, contributing to menstrual irregularities such as heavy menstrual bleeding, as observed in their case study of 13-year-old monozygotic twins. Taken together, these hypotheses suggest that methylphenidate may influence menstrual function by disrupting hormonal signalling pathways involved in cycle regulation. However, given the very small sample sizes and lack of statistical control in these case studies, these findings should be interpreted with caution, as no causal conclusions can be drawn.

Beyond menstrual health difficulties, typically-cycling individuals with ADHD diagnoses and symptoms tended to experience worsening of their ADHD symptoms during the luteal phase, as well as during menstruation.^50,51^ Some individuals also reported perceived reductions in the effectiveness of their psychostimulant medication during these periods.^50,58^ It appears that evidence of phase-specific changes in symptom severity and medication response have begun to motivate the development of phase-tailored therapeutic and pharmacological strategies.^58,59^ This reflects the gradual but growing attention being given to the potential role of menstrual cycle dynamics in ADHD treatment response.

Across all studies, menstrual cycle phases, conditions, and health features differentially influenced ADHD symptom presentation, though methodologies, measures, and sample sizes varied widely. Beyond this, though these results present multiple correlations and relationships between ADHD symptoms, attentional features, and menstrual health features, the directionality of those relationships, as well as the causal mechanisms linking them, were not established.

### Inattention in context

A key finding from our review was the consistent role of menstrual cycle features in inattention. Menstrual cycle-related fluctuations in inattention were observed among individuals diagnosed with ADHD,^50^ in community samples studied within the context of ADHD across the cycle,^51^ and in menstruators examined outside the context of ADHD.^57^ Inattention is a core symptom for many individuals AFAB with ADHD, though this symptoms does not manifest in all presentations.^36,37^ Beyond this, individuals AFAB are historically underdiagnosed with ADHD in childhood,^36,37^ in part due to greater expression of internalized symptoms, like inattention.^73–75^ For example, individuals AMAB, who tend to exhibit primarily hyperactive-impulsive and other externalizing symptoms,^73,74^ are four times more likely to be diagnosed with ADHD in childhood than individuals AFAB.^8^ However, in adulthood, this diagnosis ratio equalizes (1:1), suggesting that underdiagnosis in individuals AFAB may be due to overlooked symptom presentations, hormonal differences, or increases in symptomatology over time, rather than true prevalence differences.^8,36^ This childhood diagnosis ratio has contributed to the misconception that ADHD is less common in individuals AFAB,^36^ leading to significant gaps in research on their experiences.^36,37^

Even within the limited literature on individuals AFAB with ADHD, research has primarily focused on developmental trajectories of inattention over time.^10–12^ However, as previously mentioned, sex-specific hormonal fluctuations in menstruating individuals AFAB, like the menstrual cycle, may also contribute to shorter-term variations in symptoms. Despite this, how menstrual cycle dynamics relate to inattention remains largely unexplored. The repeated emergence of this symptom across many studies in this review, regardless of design, population, or objective, underscores the importance of understanding the mechanisms linking attentional interference to various menstrual health features, including cycle phase, pain, and bleeding severity, among other factors.

We propose that the underrepresentation of sex-specific ADHD presentations, particularly inattention, perpetuates a mischaracterization of the disorder in individuals AFAB and, in turn, contributes to their underdiagnosis. This underdiagnosis then misrepresents the true prevalence of ADHD in individuals AFAB, reinforcing the assumption that they are less affected by the condition, and therefore need not be equally represented in research. The cycle thus sustains itself: limited representation leads to mischaracterization, which leads to further exclusion. Breaking this cycle by expanding the literature on sex-specific symptom presentations is essential to improving early and accurate diagnosis and treatment in individuals AFAB.^36,37^

### Methodological and reporting limitations

For the majority, the quality of the included studies ranged from “Good” to “Very Good” (65%). However, certain methodological and reporting limitations were present. Only 5 studies (25%) presented a sample size justification based on a power analysis (quantitative) or data saturation (qualitative). Including these variables in the design and reporting of a study is important as it demonstrates that the study’s sample size was intentionally designed to detect meaningful effects or to fully capture the range of participant experiences. Beyond this, it is incredibly important to interpret the many case studies in the literature with extreme caution. Underpowered or undersaturated samples risk overinterpretation from small or biased samples, potentially reinforcing misrepresentations about menstrual-related symptom variability or leading to ineffective interventions. While the existing literature is methodologically and geographically diverse, a notable strength, it also lacks cohesion in its measures, objectives, and study designs, which limits comparability across findings. Many findings also come from specific research groups, which, although encouraging, limits the generalizability of the evidence without independent replication.

Beyond this, only two studies (10%) reported the gender identity of their participants or considered it in their analyses and interpretations. Despite this, many studies referred to their participants as “women” rather than “females” or “individuals AFAB.” There is a well-established body of literature examining the intersection between gender diversity and ADHD,^76^ as well as evidence that menstruation can influence gender dysphoria.^77^ Transgender and gender-diverse individuals have also been historically underrepresented in neurodevelopmental research, despite the documented impact of minority stress on neurodevelopment and well-being.^76,78^ For these reasons, it is crucial that future studies report and account for how gender, beyond sex, shapes experiences of ADHD and menstruation.

### Review strengths

There also exist several strengths and limitations to the current review. To the authors’ knowledge, this is the first scoping review to examine the influence of diverse menstrual health factors on ADHD symptoms in individuals with and without diagnoses. It is also the first to assess the quality of this literature and to synthesize and thematically map the potential links between menstrual health and ADHD.

### Review limitations

Some limitations should be noted. As quantitative researchers, our Quality Assessment criteria may not have fully captured the diversity and methodological nuances of the included qualitative research. To minimize potential bias, we automatically awarded points to qualitative studies when standard criteria were not applicable and incorporated qualitative-specific indicators into our adapted Risk of Bias (Quality Assessment) form. Beyond this, the Quality Assessment point system was designed to prioritize studies that directly assessed ADHD symptoms or diagnoses. We included certain studies^39,56,57^ that examined menstrual cycle-related inattention and impulsivity outside of the context of ADHD to provide a broader understanding of the literature, considering the limited research available on this subject. However, these studies received lower scores on the Quality Assessment criteria due to their lack of relevance to ADHD specifically, which may not accurately represent the depth and quality of the studies themselves.

Given that this is an emerging research area, it is also possible that new studies have been prepared, accepted, or published since the search was completed on July 18^th^, 2025. This highlights the importance of updating and replicating this scoping review over time to capture the evolving and increasingly nuanced body of literature on this topic.

### Future directions

This scoping review demonstrates that there are clear links between menstrual health and ADHD symptom variability, but methodological limitations and limited knowledge about the biopsychosocial mechanisms underlying these links constrain firm conclusions.

Future work should focus on exploring the neurological, hormonal, lifestyle, and broader societal contributors to experiences of ADHD throughout the menstrual cycle. Methodologically, it is crucial that future studies use diverse, well-justified samples and gender-sensitive approaches. In studying clinical populations, psychiatrist-confirmed or scale-validated self-reported ADHD diagnoses should also become standard practice.

While it is incredibly encouraging that some research groups are investigating menstrual health in the context of ADHD, replication by independent teams will be essential to confirm these findings across diverse geographical populations. It will also be crucial to investigate the influence of birth control on ADHD symptoms. Future research should include and compare individuals using different forms of hormonal and non-hormonal contraception, ensuring that distinctions between specific progestin types are explicitly analyzed and reported (e.g., various oral contraceptive brands, copper and hormonal intrauterine devices, implants, etc.).

Finally, intervention trials will be critical to improving care for menstruating individuals with ADHD. The preliminary evidence summarized in this review offers an important foundation, but randomized controlled trials are ultimately necessary to establish causal links and inform evidence-based, cycle-informed clinical practices.

## Conclusion

This growing field has the potential to transform how ADHD is conceptualized and managed by recognizing symptom variability as dynamic rather than static. Given the field’s history of defining ADHD through predominantly male presentations, menstrual health offers a lens to recontextualize what ADHD looks like and who it affects. With continued theoretical and methodological rigor, this work has the potential to reshape how researchers, clinicians, and systems understand and support menstruating individuals with ADHD.

## Supplemental material

Supplemental material - Menstrual health and Attention-Deficit/Hyperactivity Disorder (ADHD) symptoms: A scoping review Supplemental material for Menstrual health and Attention-Deficit/Hyperactivity Disorder (ADHD) symptoms: A scoping review by Gabriela Kennedy, Megan Baran-Goldwax, and Sarah Lippé in Women’s Health.

Supplemental material - Menstrual health and Attention-Deficit/Hyperactivity Disorder (ADHD) symptoms: A scoping review Supplemental material for Menstrual health and Attention-Deficit/Hyperactivity Disorder (ADHD) symptoms: A scoping review by Gabriela Kennedy, Megan Baran-Goldwax, and Sarah Lippé in Women’s Health.

Supplemental material - Menstrual health and Attention-Deficit/Hyperactivity Disorder (ADHD) symptoms: A scoping review Supplemental material for Menstrual health and Attention-Deficit/Hyperactivity Disorder (ADHD) symptoms: A scoping review by Gabriela Kennedy, Megan Baran-Goldwax, and Sarah Lippé in Women’s Health.

Supplemental material - Menstrual health and Attention-Deficit/Hyperactivity Disorder (ADHD) symptoms: A scoping review Supplemental material for Menstrual health and Attention-Deficit/Hyperactivity Disorder (ADHD) symptoms: A scoping review by Gabriela Kennedy, Megan Baran-Goldwax, and Sarah Lippé in Women’s Health.

Supplemental material - Menstrual health and Attention-Deficit/Hyperactivity Disorder (ADHD) symptoms: A scoping review Supplemental material for Menstrual health and Attention-Deficit/Hyperactivity Disorder (ADHD) symptoms: A scoping review by Gabriela Kennedy, Megan Baran-Goldwax, and Sarah Lippé in Women’s Health.

Supplemental material - Menstrual health and Attention-Deficit/Hyperactivity Disorder (ADHD) symptoms: A scoping review Supplemental material for Menstrual health and Attention-Deficit/Hyperactivity Disorder (ADHD) symptoms: A scoping review by Gabriela Kennedy, Megan Baran-Goldwax, and Sarah Lippé in Women’s Health.

Supplemental material - Menstrual health and Attention-Deficit/Hyperactivity Disorder (ADHD) symptoms: A scoping review Supplemental material for Menstrual health and Attention-Deficit/Hyperactivity Disorder (ADHD) symptoms: A scoping review by Gabriela Kennedy, Megan Baran-Goldwax, and Sarah Lippé in Women’s Health.

Supplemental material - Menstrual health and Attention-Deficit/Hyperactivity Disorder (ADHD) symptoms: A scoping review Supplemental material for Menstrual health and Attention-Deficit/Hyperactivity Disorder (ADHD) symptoms: A scoping review by Gabriela Kennedy, Megan Baran-Goldwax, and Sarah Lippé in Women’s Health.

## Data Availability

All materials used to conduct this scoping review (e.g., search strategy, eligibility criteria, data extraction template, and quality assessment form) are provided in the supplementary materials. All data analyzed were extracted from published studies that are publicly available.*
