# Supplementary material for: Menstrual health and Attention-Deficit/Hyperactivity Disorder (ADHD) symptoms: A scoping review
Source: Womens Health (Lond). 2026 Jun 11;22:17455057261460285. doi: 10.1177/17455057261460285 (PMC13260955; doi:10.1177/17455057261460285)
Supplement: Supplemental material - Menstrual health and Attention-Deficit/Hyperactivity Disorder (ADHD) symptoms: A scoping review [file sj-pdf-2-whe-10.1177_17455057261460285.pdf]

Supplementary Table 1. *Study Characteristics*

Study	Study Design	Method	Setting	Geographical Region	n	Age	Gender Identity	ADHD Variable	Menstrual Variable	Bias Score	Relevant Results
Comorbid ADHD Symptoms in Individuals with Menstruation-Related Conditions (n = 5)											
Ko 2024	Non-randomised experimental study	Self-report measures and saliva sampling	Community	Taiwan	108 (58 PMDD, 50 control)	Range N/A (M = 28.09, SD = 4.72 [PMDD group]; M = 27.98, SD = 4.83 [controls])	N/A	Symptoms	Menstrual Phase, PMDD	9	Women with PMDD experienced significantly worse PMDD symptoms during the late luteal (LL) phase (depression, perceived stress, inattention, craving for sweet foods, fatigue). The exacerbation of PMDD symptoms during the LL phase was associated with elevated progesterone across the luteal phase, particularly during the mid- and late-luteal phases, among women with PMDD. Vascular endothelial growth factor (VEGF) levels were negatively correlated with inattention among all the participants
Lin et al., 2021	Non-randomised experimental study	Self-report measures	Community	Taiwan	196 (100 PMDD, 96 control)	Range N/A (M = 24.77, SD = 3.32 [PMDD group]; M = 24.84, SD = 3.46 [controls])	N/A	Symptoms	PMDD	9	Women with PMDD reported greater insomnia, inattentiveness, and fatigue than controls in both the premenstrual and follicular phases, with higher symptom severity in the luteal vs. follicular phase. A significant PMDD × menstrual cycle phase interaction was observed for all symptoms, which were positively correlated with PMDD-related functional impairment.
Lin et al., 2022	Non-randomised experimental study	Self-report measures	Community	Taiwan	117 (63 PMDD, 54 control)	Range N/A (M = 25.02, SD = 3.51 [PMDD group]; M = 24.98, SD = 3.73	N/A	Symptoms	Menstrual Phase, PMDD	9	Women with PMDD showed greater inattention and poorer executive functioning during the early and late luteal phases. In the LL phase, inattention, insomnia, fatigue, and reappraisal were significantly

						[controls])					correlated with PMDD-related functional impairment, with logistic regression identifying inattention (followed by fatigue) as the strongest associated factors.
Lin et al., 2024	Non-randomised experimental study	Self-report measures	Community	Taiwan	108 (58 PMDD/ADHD, 50 control)	Range N/A (M = 28.09, SD = 4.72 [PMDD/ADHD group]; M = 27.98, SD = 4.83 [controls])	N/A	Symptoms	Menstrual Phase, PMDD	9	Women with PMDD were more likely to have comorbid ADHD (27.6%) and reported greater inattention and prospective everyday memory problems across the menstrual cycle, particularly in the post-ovulatory and LL phases. PMDD was associated with greater difficulty maintaining focused attention and higher impulsivity in the LL phase, with significant PMDD × menstrual cycle interactions for attention and impulsivity. However, impulsivity elevations were not persistent outside the context of ADHD.
Hergüner 2015	Cross sectional study	Self-report measures and blood sampling	Clinic	Turkey	80	18-35 (M = 22.28 [PCOS group], M = 22.33 [controls])	N/A	Symptoms	PCOS	9	Women with PCOS had significantly higher hyperactivity-impulsivity but not inattention scores than controls, as well as higher ADHD symptom scores (both inattentive and hyperactive/impulsive) compared to healthy controls.
Dysmenorrhea, Menorrhagia, Irregular Menstruation and ADHD Symptoms (n = 4)											
Kabukcu et al., 2021	Cross sectional study	Self-report measures	Clinic	Turkey	209	13-18 (M = 15.47)	N/A	Symptoms	Dysmenorrhea	8	Adolescents with dysmenorrhea affecting daily life had more symptoms of inattention and hyperactivity-impulsivity, as well as other psychological symptoms (anxiety, depression, somatization, negative self-perception, hostility). As the severity of menstrual pain increased, the severity of ADHD symptoms increased.

Lockinger & Gagnon, 2023	Cross sectional study	Self-report measures	Community	Canada	266	18-51(M = 30.94, SD = 6.59)	Cisgender/transgender man: 2 (0.8%) Cisgender/transgender woman: 237 (89.1%) Non-Binary: 22 (8.3%) Other: 2 (0.8%)	Symptoms	Dysmenorrhea	10	ADHD symptom severity was moderately and positively correlated with dysmenorrhea severity, with a high comorbidity rate (~95%), stronger than associations with anxiety or depression. ADHD symptom severity was also negatively correlated with psychological well-being, and emotion regulation did not significantly moderate these relationships.
MacLean et al., 2025	Cross sectional study	Self-report measures and blood sampling	Community	Australia	405	18-49 (M = 24.8, SD = 10.1)	N/A	Symptoms	Menorrhagia	10	ADHD symptoms were reported by 43% of participants and were associated with a higher prevalence of heavy menstrual bleeding (39.1% vs. 26.0%). Women with ADHD symptoms also reported more frequent iron-deficiency-type symptoms (e.g., fatigue, dizziness, brain fog, anxiety, palpitations), although anaemia status was not significantly associated with ADHD symptoms.
Yuan et al., 2024	Other: Longitudinal Study	Self-report measures	School	China	Baseline = 1039, 1-year follow-up = 946	4-8th grade (M = 12.49, SD = 0.97)	N/A	Symptoms	Menstrual Cycle Regularity	8	No direct link was found between menstrual irregularity and ADHD, though irregular cycles may contribute to symptom maintenance. Early menarche and menstrual irregularities were associated with higher rates of anxiety, insomnia, depression, and other mental health problems, while menstrual pain was linked to elevated rates of PTSD, depression, and self-harm behaviors.
Fluctuations in ADHD Symptoms Throughout the Menstrual Cycle (n = 4)											
Bürger et al., 2024	Qualitative research	Semi-structured qualitative interviews	Community	Sweden	10	23-39 (M = N/A, median = 27)	Women and non-binary, percentages N/A	Dx	Menstrual phase	5	ADHD symptoms worsened during the mid-luteal phase and menses. Symptoms included executive

											dysfunction (task switching, task initiation), emotional dysregulation (irritability, sadness, anxiety, agitation, sensitivity, hopelessness), and attention dysregulation, (concentration, organization, staying focused, forgetfulness)
Roberts et al., 2018	Non-randomised experimental study	Self-report and saliva sampling	Community	United States	32	18-22 (M = 19.43, SD = 1.38)	N/A	Symptoms	Menstrual Phase	10	Higher average estradiol (E2) and progesterone (P4) predicted lower inattention, with P4 variability also linked to reduced inattention. Hormonal effects on inattention and hyperactivity were strongest in women high in Sensation Seeking or Urgency traits. Post-ovulatory and follicular phases showed elevated symptoms in these women, suggesting ADHD symptoms may fluctuate within women and be influenced by hormones and personality.
Zhuang et al., 2020 **Study A**	Non-randomised experimental study	Physiological measures (fMRI tasks)	Community	China	24	20-25 (M = 22.79, SD = 1.44)	N/A	Symptoms	Menstrual Phase	7	Women showed higher impulsivity in the late follicular vs. mid-luteal phase, linked to increased dorsal striatum activity. Dorsal striatum - dorsolateral prefrontal cortex (DS-dlPFC) connectivity modulated impulsivity across phases, suggesting stronger cognitive control in the mid-luteal phase.
Zhuang et al., 2020 **Study A**	Non-randomised experimental study	Self-report, saliva sampling and physiological measures (rs-fMRI)	Community	China	53	19-28 (M = 22.77, SD = 2.35)	N/A	Symptoms	Menstrual Phase	9	Hormone assays confirmed menstrual phases. Resting state fMRI showed greater right dlPFC activity during the mid-luteal phase, linked to stronger cognitive control. DS-dlPFC connectivity correlated negatively with attentional impulsivity in the late follicular phase. Cognitive control was associated with relative estradiol and progesterone levels, while social

											cognition regions were more sensitive to progesterone during the mid-luteal phase.
Osmotic-Release Oral System Methylphenidate (OROS-MPH) and Menstrual Side-Effects (n = 3)											
Coskun & Adak, 2017	Case report	Self-report and medical examination	Clinic	Turkey	1	17	N/A	Dx	Hypermenorrhea and Polymenorrhea	2	Link between Osmotic-Release Oral System Methylphenidate (OROS-MPH) and excessive and frequent menstrual bleeding in one adolescent. Resolved following prescription of oral contraceptive.
Mutlu et al., 2016	Case report	Self-report and medical examination	Clinic	Turkey	1	17	N/A	Dx	Menstrual Cycle Length	2	Increased doses of OROS-MPH were associated with prolonged menstrual cycle length in one adolescent. Reducing the dose to 27 mg/day resolved the menstrual abnormality. This case suggests a possible dose-related effect of methylphenidate on menstrual cycle length.
Ozdag et al., 2022	Case report	Self-report and medical examination	Clinic	Turkey	2	13	N/A	Dx	Menorrhagia	2	Two adolescent identical twins experienced increased menstrual cycle length and menorrhagia after starting methylphenidate monotherapy. Both discontinued the medication, after which their periods returned to normal. Clinical evaluation excluded other causes.
Menstrual Pain-Related Attentional Interference (n = 2)											
Aziato et al., 2014	Qualitative research	Semi-structured qualitative interviews	School	Ghana	16	16-38 (M = N/A)	N/A	Pain-related inattention	Dysmenorrhea	5	Dysmenorrhea was associated with absenteeism and inattentiveness in class. Symptoms disrupted daily activities, impaired concentration, altered mood and perpetuated misconceptions about menstruation.
Keogh 2014	Non-randomised experimental	Self-report measures and behavioural	Community	United Kingdom	52	18-46 (M = 22.09)	N/A	Pain-related inattention	Menstrual Pain	6	Greater attentional interference effects (worse performance) were found to occur during the menstrual

	study	measures (attentional interference tasks)									pain phase compared to the non-pain phase (responding slower, false alarms, less accuracy).
Menstrual Cycle-Tailored Interventions for Individuals with ADHD and Menstrual Symptom Variation (n = 2)											
De Jong et al., 2024	Qualitative research	Self-report measures and seven bi-weekly group therapy sessions	Clinic	Netherlands	18	25-47 (M = 37.8)	N/A	Symptoms	Menstrual Phase	7	Female-specific psychoeducation combined with individualized, cycle-informed treatment may enhance adherence and clinical outcomes in women with ADHD who experience pre-menstrual worsening in their symptoms.
De Jong et al., 2023	Case series	Self-report and medical examination	Clinic	Netherlands	9	22-48 (M = N/A)	N/A	Dx	Menstrual Phase	3	Increasing psychostimulant dose premenstrually resulted in a positive change in premenstrual mood (more energy, less irritability) and ADHD symptoms (concentration, focus, productivity, emotion regulation).

Note. Summary of the characteristics of the 20 studies included in this review grouped by theme. Columns report the study name, design, methodology, setting, geographical region, sample size (n), participant age, gender identity, ADHD-related variables assessed, menstrual-related variables assessed, bias score, and key results relevant to ADHD and menstrual health.
