# Supplementary material for: Menstrual health and Attention-Deficit/Hyperactivity Disorder (ADHD) symptoms: A scoping review
Source: Womens Health (Lond). 2026 Jun 11;22:17455057261460285. doi: 10.1177/17455057261460285 (PMC13260955; doi:10.1177/17455057261460285)
Supplement: Supplemental material - Menstrual health and Attention-Deficit/Hyperactivity Disorder (ADHD) symptoms: A scoping review [file sj-pdf-3-whe-10.1177_17455057261460285.pdf]

Appendix I

Eligibility Criteria

Inclusion Criteria	Exclusion Criteria
**Population**	
• Individuals assigned female at birth who menstruate<br>• Adolescents or adults with ADHD diagnosis or who present ADHD symptoms at any point in their menstrual cycle (inattention, impulsivity, hyperactivity)<br>• Any diagnostic method (clinical diagnosis, self-report, validated scale, brain activity)	• Pre-menarcheal individuals<br>• Postmenopausal individuals<br>• Individuals who do not present ADHD symptoms at any point in their menstrual cycle<br>• Studies focusing solely on other neurodevelopmental or psychiatric conditions (e.g., Autism, depression, anxiety) without ADHD symptom data
**Intervention / Exposure**	
• Menstrual cycle phases (e.g., follicular, ovulatory, luteal, menstrual)<br>• Menstrual bleeding / Heaviness of menstrual bleed<br>• Timing / dosage of psychostimulant medication across menstrual cycle<br>• Within-cycle variation (explicit reference to cycle timing or phase)	• Exogenous hormone interventions (e.g., hormone therapy, contraceptive use) unless menstrual cycle phase or hormonal timing is explicitly analyzed<br>• Menstrual-related interventions (e.g., period tracking apps, exercise, therapy) with no ADHD outcome data
**Comparator / Context**	
• Any or none (comparison groups not required)<br>• Within-subject comparisons across menstrual phases	• Studies only comparing ADHD and non-ADHD groups outside of a menstrual cycle framework
**Outcome**	
• ADHD symptom expression (inattention, hyperactivity, impulsivity)<br>• Measured through self-report, clinical assessment, observational data, or standardized tools	• Studies focusing on mood, anxiety, emotional regulation, or other psychiatric symptoms in individuals with ADHD<br>• Cognitive outcomes not directly linked to ADHD symptoms (e.g., memory, IQ without ADHD framing)

Study Characteristics	
• Human studies<br>• Peer-reviewed journal articles<br>• All study designs (e.g., qualitative, quantitative, mixed methods)	• Reviews, commentaries, editorials (can be used for background, not for scoping data extraction)<br>• Animal studies<br>• Unpublished theses<br>• Conference abstracts
**Other**	
• Any geographic location<br>• Any publication year<br>• English or French	• Duplicates
