# Supplementary material for: Menstrual health and Attention-Deficit/Hyperactivity Disorder (ADHD) symptoms: A scoping review
Source: Womens Health (Lond). 2026 Jun 11;22:17455057261460285. doi: 10.1177/17455057261460285 (PMC13260955; doi:10.1177/17455057261460285)
Supplement: Supplemental material - Menstrual health and Attention-Deficit/Hyperactivity Disorder (ADHD) symptoms: A scoping review [file sj-pdf-4-whe-10.1177_17455057261460285.pdf]

Appendix II

Search Strategy

The following search terms were used for a search conducted on MEDLINE (via Ovid), PsycINFO (via Ovid), and Web of Science on the 18th of July, 2025. No limits were placed on date, language was limited to French and English:

ADHD or hyperactiv* or "attention deficit" or inattent*

AND

menstrua* or premenstrua* or perimenstrua* or ovulat* or menorrh* or dysmenorrh* or amenorrh* ot "ovar* cycle*" or "ovul* cycle*" or "ovar* period*" or "ovul* period*" or menses
