# Supplementary material for: Menstrual health and Attention-Deficit/Hyperactivity Disorder (ADHD) symptoms: A scoping review
Source: Womens Health (Lond). 2026 Jun 11;22:17455057261460285. doi: 10.1177/17455057261460285 (PMC13260955; doi:10.1177/17455057261460285)
Supplement: Supplemental material - Menstrual health and Attention-Deficit/Hyperactivity Disorder (ADHD) symptoms: A scoping review [file sj-pdf-5-whe-10.1177_17455057261460285.pdf]

Appendix III

Data Extraction Template (adapted from Covidence)

General information

Study ID:

DOI:

Title:

Title of paper / abstract / report that data are extracted from

Authors:

Journal:

Publication Date:

Country in which the study conducted:

- United States
- UK
- Canada
- Australia
- Other:

Characteristics of included studies

Methods

Aim of study:

Study design:

- Randomised controlled trial
- Non-randomised experimental study
- Cohort study

- Cross sectional study
- Case control study
- Systematic review
- Qualitative research
- Prevalence study
- Case series
- Case report
- Diagnostic test accuracy study
- Clinical prediction rule
- Economic evaluation
- Text and opinion
- Other

Participants

Sample Size (Total N):

Population type (e.g., adults, adolescents, clinical diagnosis):

Age Range:

If unavailable, indicate N/A

Mean Age:

If unavailable, indicate N/A

Sex Distribution:

If unavailable, indicate N/A

Gender Distribution:

If unavailable, indicate N/A

Inclusion Criteria:

Exclusion Criteria:

Concept(s) Examined

Exposure (Predictor) (ex: Menstrual cycle phase, psychostimulant use):

Outcome (Result) (ex: ADHD symptoms, cognitive performance, menstrual bleeding patterns):

Context

Setting (e.g., school, community, clinic):

Geographical/cultural context:

If relevant/discussed. If not, indicate N/A.

Methods/measures

Tools or instruments used:

Menstrual cycle-related variables measured (phase, hormone assays, self-report, etc.):

ADHD-related variables included (self-report, clinical diagnosis, cognitive tests, etc.):

Timing/frequency of data collection:

Key Findings

Summary of results directly related to research questions:

Author-reported conclusions:

Reviewer-Identified Emerging Theme

Review themes that emerge:

Based on the study's findings, note any concise theme or concept that may appear across multiple studies. Use brief, descriptive titles (e.g., "X symptom worsening pre-menses," "Stimulant dosage adjustment by cycle phase"). This is a preliminary coding step for later synthesis and thematic grouping.

Notes/Other:

Reviewer notes (e.g., methodological limitations, anything unusual):

