# Supplementary material for: Menstrual health and Attention-Deficit/Hyperactivity Disorder (ADHD) symptoms: A scoping review
Source: Womens Health (Lond). 2026 Jun 11;22:17455057261460285. doi: 10.1177/17455057261460285 (PMC13260955; doi:10.1177/17455057261460285)
Supplement: Supplemental material - Menstrual health and Attention-Deficit/Hyperactivity Disorder (ADHD) symptoms: A scoping review [file sj-pdf-6-whe-10.1177_17455057261460285.pdf]

Appendix IV

Quality Assessment Form/Risk of Bias Criteria

Risk of Bias Criteria - Adapted Version (GK & MBG)

Source: https://doi.org/10.1177/10870547251332319

Selection (Maximum 5 stars)

1. **Representativeness of the Exposed Cohort**
   a. Truly representative of average in target population * (all subjects or random sampling)
   b. Somewhat representative of average in target population * (non-random sampling)
   c. Selected group of users
   d. No description of sampling strategy
2. **Sample size justification**
   a. Justification of sample size based on power analysis (quantitative) or based on data saturation (qualitative)*
   b. Not justified
3. **Exclusion or inclusion criteria mentioned**
   a. Inclusion **and** exclusion criteria mentioned*
   b. Inclusion **or** exclusion criteria, but clearly discernible*
   c. No mention or unclear description of inclusion and/or exclusion criteria
4. **Ascertainment of ADHD diagnosis or symptoms**
   a. ADHD diagnosis *
   b. Structure interview/ scale scores *
   c. Self-report
   d. No description
5. **Ascertainment of menstrual cycle phase or health characteristics**
   a. Details of menstrual health or phase assessment tools provided (e.g. validated measure of menstrual cycle phase/symptoms)*
   b. No details/ validation of menstrual characteristics provided

Comparability (Maximum 2 stars)

1. **Comparability of cohorts on the basis of the design or analysis**
   a. Study controls for most important factor (specific relevant variables related to ADHD/menstruation - ADHD medication usage, hormonal disorders/treatment use, etc.)*
   b. The study controls for any additional factor or is qualitative in nature (demographic variables such as age, socioeconomic status, gender, diagnostic information, and other study-specific characteristics)*
   c. Inadequate degree of control

Outcome (Maximum 3 stars)

1. **Assessment of outcome**
   a. Validated method to measure ADHD symptoms **
   b. Non validated measurement tool, but method is available or described *
   c. No description of measurement tool
   d. Does not measure ADHD symptoms
2. **Statistical test**
   a. Test used to analyse data clearly described and appropriate, and measurement of association is presented, including probability level (p value)*
   b. No statistical test, or the test in not appropriate, not described, or incomplete

Total score: (Possible scores 0-10)

Scoring (from article):

- 0 to 4 = Unsatisfactory
- 5 to 6 = Satisfactory
- 7 to 8 = Good
- 9 to 10 = Very Good

*Areas highlighted in pink are modified questions/wording.
