# Supplementary material for: Menstrual health and Attention-Deficit/Hyperactivity Disorder (ADHD) symptoms: A scoping review
Source: Womens Health (Lond). 2026 Jun 11;22:17455057261460285. doi: 10.1177/17455057261460285 (PMC13260955; doi:10.1177/17455057261460285)
Supplement: Supplemental material - Menstrual health and Attention-Deficit/Hyperactivity Disorder (ADHD) symptoms: A scoping review [file sj-pdf-7-whe-10.1177_17455057261460285.pdf]

Appendix V

Quality Assessment Results

Study ID	Title	Score	Representativeness of the exposed cohort (1 star max)	Sample size justification (1 star max)	Inclusion or exclusion criteria mentioned (1 star max)	Ascertainment of ADHD diagnosis or symptoms (1 star max)	Ascertainment of menstrual cycle phase or health characteristics (1 star max)	Comparability of cohorts on the basis of the design or analysis (2 stars max)	Assessment of outcome (2 stars max)	Statistical test (1 star max)
Keogh 2014	The effects of menstrual-related pain on attentional interference.	6	Truly representative of average in target population * (all subjects or random sampling)	Not justified	Inclusion and exclusion criteria mentioned*	No description	Details of menstrual health or phase assessment tools provided*<br><br>(Self-reported menstrual cycle phases, home ovulation detection kits)	The study controls for any additional factor or is qualitative in nature**	Does not measure ADHD symptoms<br><br>(Pain-related behavioural inattention tasks used, unrelated to ADHD symptomology)	Test used to analyse data clearly described and appropriate, and measurement of association is presented, including probability level (p value) (unless qualitative)*
Aziato 2014	The experience of dysmenorrhoea among Ghanaian senior high and university students: pain characteristics and effects.	6	Selected group of users	Justification of sample size based on power analysis (quantitative) or based on data saturation (qualitative) *	Inclusion or exclusion criteria mentioned, but criteria clearly discernible*	Self-report<br><br>(Inattentiveness/ absenteeism emerged as qualitative theme)	Details of menstrual health or phase assessment tools provided*<br><br>(Self-reported onset and duration of dysmenorrhoea relative to menstruation, pain characteristics and timing of symptoms in relation to menstrual cycle phases)	The study controls for any additional factor or is qualitative in nature**	Does not measure ADHD symptoms<br><br>(Grouped inattentiveness/ absenteeism as one theme)	Test used to analyse data clearly described and appropriate, and measurement of association is presented, including probability level (p value) (unless qualitative)*
Hergüner 2015	Attention deficit-hyperactivity disorder symptoms in women with	9	Somewhat representative of average in target population * (non-random sampling)	Not justified	Inclusion and exclusion criteria mentioned*	Structure interview / scale scores (Adult ADHD Self-Report Scale,	Details of menstrual health or phase assessment tools provided*	Study controls for most important factor**	Validated method to measure ADHD symptoms **	Test used to analyse data clearly described and appropriate, and measurement of association is

	polycystic ovary syndrome.					Wender-Utah Rating Scale)*	(Blood samples of hormone levels)	(PCOS diagnosis, hormone levels)		presented, including probability level (p value) (unless qualitative)*
Mutlu 2016	Increase in Menstrual Cycle Length Induced by Extended-Release Methylphenidate in an Adolescent with Attention-Deficit/Hyperactivity Disorder.	2	Selected group of users	Not justified	No mention or unclear description of inclusion and/or exclusion criteria	ADHD diagnosis<br><br>(Clinician assessed diagnosis and symptoms) *	Details of menstrual health or phase assessment tools provided<br><br>(Self-reported cycle length to clinician)*	Inadequate degree of control	No description of measurement tool	No statistical test, or the test in not appropriate, not described, or incomplete
Coskun 2017	Excessive and Frequent Menstrual Bleeding With Methylphenidate in an Adolescent Girl With Attention-Deficit Hyperactivity Disorder.	1	Selected group of users	Not justified	No mention or unclear description of inclusion and/or exclusion criteria	ADHD diagnosis *	No details / validation of menstrual characteristics provided	Inadequate degree of control	No description of measurement tool	No statistical test, or the test in not appropriate, not described, or incomplete
Roberts 2018	Reproductive steroids and ADHD symptoms across the menstrual cycle.	10	Truly representative of average in target population *<br><br>(All subjects or random sampling)	Justification of sample size based on power analysis<br><br>(Quantitative) or based on data saturation (qualitative) *	Inclusion or exclusion criteria mentioned, but criteria clearly discernible*	Structure interview / scale scores<br><br>(Self-reported UPPS-P Trait Impulsivity Scale and Current	Details of menstrual health or phase assessment tools provided*<br><br>(Saliva samples for hormone levels every other day)	The study controls for any additional factor or is qualitative in nature**	Validated method to measure ADHD symptoms**	Test used to analyse data clearly described and appropriate, and measurement of association is presented, including probability level (p value) (unless qualitative)*

						ADHD Symptoms Scale) *				
Kabukcu 2021	Primary dysmenorrhea in adolescents: Association with attention deficit hyperactivity disorder and psychological symptoms.	8	Somewhat representative of average in target population * (Non-random sampling)	Not justified	No mention or unclear description of inclusion and/or exclusion criteria	Structure interview / scale scores (Self-reported ADHD symptoms using T-DSM-IV-S scale)*	Details of menstrual health or phase assessment tools provided* (In-house questionnaire for sociodemographic data, menstrual pattern, and dysmenorrhea)	Study controls for most important factor** (Dysmenorrhea, sleep, behavioural and psychological symptoms)	Validated method to measure ADHD symptoms**	Test used to analyse data clearly described and appropriate, and measurement of association is presented, including probability level (p value) (unless qualitative)*
Lin 2021	Insomnia, Inattention and Fatigue Symptoms of Women with Premenstrual Dysphoric Disorder.	9	Truly representative of average in target population * (All subjects or random sampling)	Not justified	Inclusion and exclusion criteria mentioned*	Structure interview / scale scores (Inattention symptoms measured with Attention and Performance Self-Assessment Scale*	Details of menstrual health or phase assessment tools provided* (Self-reported menstrual cycle tracking)	Study controls for most important factor** (PMDD diagnosis)	Validated method to measure ADHD symptoms**	Test used to analyse data clearly described and appropriate, and measurement of association is presented, including probability level (p value) (unless qualitative)*
Zhuang 2020	**Study A** Neural Basis of Increased Cognitive Control of Impulsivity During the Mid-Luteal Phase Relative to the Late Follicular Phase of the	6	Somewhat representative of average in target population * (Non-random sampling)	Not justified	Inclusion or exclusion criteria mentioned, but criteria clearly discernible*	No description	Details of menstrual health or phase assessment tools provided* (Self-reported menstrual phases/backwards counting)	The study controls for any additional factor or is qualitative in nature**	Does not measure ADHD symptoms (fMRI delay discounting task measuring cognitive control / impulsiveness)	Test used to analyse data clearly described and appropriate, and measurement of association is presented, including probability level (p value) (unless qualitative)*

	Menstrual Cycle.									
Zhuang 2020	**Study B** Neural Basis of Increased Cognitive Control of Impulsivity During the Mid-Luteal Phase Relative to the Late Follicular Phase of the Menstrual Cycle.	9	Somewhat representative of average in target population * (Non-random sampling)	Not justified	Inclusion and exclusion criteria mentioned*	Structure interview / scale scores (Self-reported inattentiveness symptoms on BIS-11 scale) *	Details of menstrual health or phase assessment tools provided* (Self-reported menstrual phases/backwards counting, hormone assays)	The study controls for any additional factor or is qualitative in nature**	Validated method to measure ADHD symptoms**	Test used to analyse data clearly described and appropriate, and measurement of association is presented, including probability level (p value) (unless qualitative)*
Lin 2022	Early and Late Luteal Executive Function, Cognitive and Somatic Symptoms, and Emotional Regulation of Women with Premenstrual Dysphoric Disorder.	9	Truly representative of average in target population * (All subjects or random sampling)	Not justified	Inclusion and exclusion criteria mentioned*	Structure interview / scale scores (Attention subscale in Attention and Performance Self-Assessment Scale)*	Details of menstrual health or phase assessment tools provided* (Self-reported menstrual cycle phases and symptoms, The Premenstrual Symptoms Screening Tool (PSST))	Study controls for most important factor** (PMDD diagnosis)	Validated method to measure ADHD symptoms**	Test used to analyse data clearly described and appropriate, and measurement of association is presented, including probability level (p value) (unless qualitative)*
Ozdag 2022	Methylphenidate-Induced Menorrhagia in Twin Girls.	2	Selected group of users	Not justified	No mention or unclear description of	ADHD diagnosis (Clinical assessment -	Details of menstrual health or phase assessment tools provided*	Inadequate degree of control	No description of measurement tool	No statistical test, or the test in not appropriate, not

					inclusion and/or exclusion criteria	Conner's Family and Teacher Rating Scales), self-reported symptoms*	(Self-reported pain, bleeding and cycle information)			described, or incomplete
de Jong 2023	Female-specific pharmacotherapy in ADHD: premenstrual adjustment of psychostimulant dosage.	3	Selected group of users	Not justified	No mention or unclear description of inclusion and/or exclusion criteria	ADHD diagnosis<br><br>(Confirmed with DIVA-5 interview)*	Details of menstrual health or phase assessment tools provided*<br><br>(Self-reported premenstrual phase and related mood symptoms)	Inadequate degree of control	Non validated measurement tool, but method is available or described*	No statistical test, or the test in not appropriate, not described, or incomplete
de Jong 2024	A Female-Specific Treatment Group for ADHD-Description of the Programme and Qualitative Analysis of First Experiences.	7	Selected group of users	Not justified	Inclusion and exclusion criteria mentioned*	ADHD diagnosis<br><br>(Confirmed with DIVA-5 interview) *	Details of menstrual health or phase assessment tools provided*<br><br>(Self-reported menstrual cycle phases and symptoms)	Study controls for most important factor**<br><br>(Active suicidality, mood symptoms)	Validated method to measure ADHD symptoms**	No statistical test, or the test in not appropriate, not described, or incomplete
Ko 2024	Estrogen, progesterone, cortisol, brain-derived neurotrophic factor, and vascular endothelial growth factor during the luteal phase of the menstrual cycle in women with	9	Truly representative of average in target population *<br><br>(All subjects or random sampling)	Not justified	Inclusion and exclusion criteria mentioned*	Structure interview / scale scores<br><br>(ASRS questionnaire) *	Details of menstrual health or phase assessment tools provided*<br><br>(Hormone assays and self-reported cycle phase and symptoms)	Study controls for most important factor**<br><br>(PMDD diagnosis, hormone levels)	Validated method to measure ADHD symptoms**	Test used to analyse data clearly described and appropriate, and measurement of association is presented, including probability level (p value) (unless qualitative)*

	premenstrual dysphoric disorder.									
Yuan 2024	Longitudinal associations of menstrual characteristics with mental health problems among Chinese girls.	8	Somewhat representative of average in target population * (Non-random sampling)	Not justified	No mention or unclear description of inclusion and/or exclusion criteria	Structure interview / scale scores (ASRS questionnaire) *	Details of menstrual health or phase assessment tools provided* (In-house self-report questionnaires on menarche, menstrual cycle, and menstrual pain)	The study controls for any additional factor or is qualitative in nature**	Validated method to measure ADHD symptoms**	Test used to analyse data clearly described and appropriate, and measurement of association is presented, including probability level (p value) (unless qualitative)*
Bürger 2024	Perceived associations between the menstrual cycle and Attention Deficit Hyperactivity Disorder (ADHD): A qualitative interview study exploring lived experiences.	9	Somewhat representative of average in target population * (Non-random sampling)	Justification of sample size based on power analysis (quantitative) or based on data saturation (qualitative) *	No mention or unclear description of inclusion and/or exclusion criteria	ADHD diagnosis *	Details of menstrual health or phase assessment tools provided* (Semi-structured interview assessing menstrual cycle symptoms across cycle phases)	The study controls for any additional factor or is qualitative in nature**	Non validated measurement tool, but method is available or described*	Test used to analyse data clearly described and appropriate, and measurement of association is presented, including probability level (p value) (unless qualitative)*
Lin 2024	Comorbid Attention Deficit Hyperactivity Disorder in Women with Premenstrual Dysphoric Disorder.	9	Somewhat representative of average in target population * (Non-random sampling)	Not justified	Inclusion and exclusion criteria mentioned*	ADHD diagnosis (Confirmed using the Mini-International Neuropsychiatric Interview to assess DSM-5	Details of menstrual health or phase assessment tools provided* (Self-reported menstrual phases validated with ovulation prediction kit)	Study controls for most important factor** (PMDD diagnosis)	Validated method to measure ADHD symptoms**	Test used to analyse data clearly described and appropriate, and measurement of association is presented, including probability level (p

						diagnosis criteria for ADHD)<br><br>+<br><br>ADHD symptoms measured with APSA and Dickman Impulsivity Inventory *				value) (unless qualitative)*
MacLean 2025	Women with Symptoms Suggestive of ADHD Are More Likely to Report Symptoms of Iron Deficiency and Heavy Menstrual Bleeding.	10	Somewhat representative of average in target population *<br><br>(Non-random sampling)	Justification of sample size based on power analysis (quantitative) or based on data saturation (qualitative) *	Inclusion or exclusion criteria mentioned, but criteria clearly discernible*	Structure interview / scale scores<br><br>(ASRS-V1.1 Screener) *	Details of menstrual health or phase assessment tools provided*<br><br>(Hemoglobin concentration taken using finger-prick testing)	Study controls for most important factor*<br><br>(Heavy menstrual bleeding and iron deficiency symptoms)	Validated method to measure ADHD symptoms**	Test used to analyse data clearly described and appropriate, and measurement of association is presented, including probability level (p value) (unless qualitative)*
Lockinger 2023	Dysmenorrhea and psychological wellbeing among females with attention deficit hyperactivity disorder.	10	Somewhat representative of average in target population *<br><br>(Non-random sampling)	Justification of sample size based on power analysis (quantitative) or based on data saturation (qualitative) *	Inclusion or exclusion criteria mentioned, but criteria clearly discernible*	ADHD diagnosis<br><br>(Inclusion criteria)<br>+<br><br>ADHD symptoms self-reported via validated questionnaire<br><br>(ASRS questionnaire) *	Details of menstrual health or phase assessment tools provided*<br><br>(Self-reported menstrual pain via validated SSS questionnaire)	Study controls for most important factor**<br><br>(ADHD medication usage)	Validated method to measure ADHD symptoms**	Test used to analyse data clearly described and appropriate, and measurement of association is presented, including probability level (p value) (unless qualitative)*
