# Supplementary material for: Menstrual health and Attention-Deficit/Hyperactivity Disorder (ADHD) symptoms: A scoping review
Source: Womens Health (Lond). 2026 Jun 11;22:17455057261460285. doi: 10.1177/17455057261460285 (PMC13260955; doi:10.1177/17455057261460285)
Supplement: Supplemental material - Menstrual health and Attention-Deficit/Hyperactivity Disorder (ADHD) symptoms: A scoping review [file sj-pdf-8-whe-10.1177_17455057261460285.pdf]

Appendix VI

Summary of Study Designs and Assessment Time Points

<u>Cross-sectional (single assessment): 7 studies (30%)</u>

- **Aziato (2014)**: 1 assessment
    - *Not directly measuring ADHD*
- **Bürger (2024)**: 1 assessment
- **Hergüner (2015)**: 1 assessment
- **de Jong (2024):** 1 qualitative evaluation session after 7 group therapy sessions
- **Kabukcu (2021)**: 1 assessment
- **Lockinger & Gagnon (2023)**: 1 assessment
- **Maclean (2025):** 1 assessment

<u>Two time-point assessments: 4 studies (20%)</u>

- **Zhuang A (2020)**: 2 in-lab visits (late follicular, mid-luteal)
    - *Not directly measuring ADHD*
- **Zhuang B (2020)**: 2 in-lab visits (late follicular, mid-luteal)
- **Yuan (2024)**: baseline and 1-year follow-up
- **Keogh (2014)**: 2 assessments (pain phase vs non-pain phase)

<u>Three time-point assessments (within-cycle): 2 studies (10%)</u>

- **Ko (2024)**: 3 in-cycle assessments (pre-ovulatory, mid-luteal, late-luteal) + weekly symptom ratings
- **Lin (2024):** 3 in-cycle assessments (pre-ovulatory, mid-luteal, late-luteal) + weekly ratings for one cycle

<u>Multiple time points across menstrual cycle (structured): 3 studies (15%)</u>

- **Lin (2021)**: 2 in-cycle assessments (late luteal, follicular) + weekly ratings for 2 cycles
- **Lin (2022)**: 2 in-cycle assessments (early luteal, late luteal) + weekly ratings for 2 cycles
- **Roberts (2018)**: 1 in-lab visit + daily saliva and symptom ratings for 35 days (repeated within-subject measures)

Longitudinal (clinical case studies): 4 studies (20%)

- **Coskun & Adak (2017)**: 7 clinician-led assessments (based on clinical needs)
- **de Jong (2023)**: repeated clinical follow-ups (6 to 24 months; variable by participant)
- **Ozdag (2022)**: case study with longitudinal clinical follow-up (no fixed time points)
- **Mutlu (2016)**: case study with multiple menstrual-cycle assessments, 2-month and additional follow-ups

Summary (n = 20 studies)

- **Cross-sectional:** 7 (35%)
- **Two timepoints:** 4 (20%)
- **Three timepoints:** 2 (10%)
- **Multiple cycles (structured):** 3 (15%)
- **Longitudinal:** 4 (20%)
